# Real-World Evaluation of the Dosage Patterns and Safety of Angiotensin Receptor-Neprilysin Inhibitor Therapy in Indian Patients With Heart Failure With Reduced Ejection Fraction: The ADD-ARNI Study Protocol

**DOI:** 10.7759/cureus.88037

**Published:** 2025-07-15

**Authors:** Sanjay Mittal, Ashwani Mehta, Kamal Sharma, P B Jayagopal, Arindam Pande, Amarnath Sugumaran, Sandesh Sawant, Senthilnathan Mohanasundaram

**Affiliations:** 1 Cardiology, Medanta Medicity Hospital, Gurugram, IND; 2 Cardiology, Sir Ganga Ram Hospital, New Delhi, IND; 3 Cardiology, SAL Hospital and Medical Institute, Ahmedabad, IND; 4 Cardiology, Lakshmi Hospital, Palakkad, IND; 5 Cardiology, Medica Superspecialty Hospital, Kolkata, IND; 6 Medical Affairs, Cipla Ltd., Mumbai, IND

**Keywords:** angiotensin receptor-neprilysin inhibitor, dosage patterns, guideline-directed medical therapy, heart failure with reduced ejection fraction, real-world evidence

## Abstract

Background: Heart failure is a burgeoning disease that imposes an enormous social and economic burden globally. It presents a significant public health challenge due to its high morbidity and mortality. In recent years, notable advancements have been made in the pharmacological treatment of heart failure with reduced ejection fraction (HFrEF), with guideline-directed medical therapy (GDMT) emerging as the cornerstone of HFrEF management. Although the angiotensin-receptor neprilysin inhibitor (ARNI), sacubitril/valsartan, is recognized as an integral component of HFrEF pharmacotherapy, variations in dose response have been observed in the Indian population. Furthermore, substantial differences exist in heart failure management practices across various types of hospitals in India. To address this paucity, the Assessing the Dosage Pattern and Demographic Characteristics in Heart Failure Reduced Ejection Fraction Patients Initiated with Angiotensin Receptor-Neprilysin Inhibitor in India (ADD-ARNI) study was designed to characterize contemporary HFrEF pharmacotherapy in India. This is a prospective, multicenter observational study to evaluate the dosage patterns, treatment adherence, and safety of ARNI therapy in HFrEF patients in India.

Methods: The ADD-ARNI study is a prospective, multicenter, observational study designed to enroll 3000 patients diagnosed with HFrEF. The study population will consist of symptomatic chronic HFrEF patients who are eligible for the initiation of sacubitril/valsartan (ARNI) as per the clinical judgement of the treating physician. The primary endpoint of the study is the dosage pattern of sacubitril/valsartan at the start as well as the end of the study. The secondary endpoints include the dosing pattern of other concomitant HFrEF medications at the start and end of the study, such as the doses of beta-blockers (BB), mineralocorticoid receptor antagonists (MRA), sodium-glucose cotransporter 2 inhibitors (SGLT2i), and diuretics, the implementation of GDMT at the end of the study, the assessment of the effectiveness of sacubitril/valsartan therapy based on changes in functional capacity and N-terminal pro-B-type natriuretic peptide (NT-proBNP) levels from baseline to follow-up, and the incidence of adverse events, either spontaneously reported by the patients or noticed by the clinician during the study.

Conclusion: In heart failure management, there is an unmet clinical need to gain insights about the real-world evidence of pharmacotherapy, adherence to current practice guidelines, and impact on clinical outcomes. Undertreatment with doses less than 50% of the guideline-recommended target doses is associated with poorer prognosis. The ADD-ARNI study will provide real-world evidence on dosage patterns, effectiveness, treatment persistence, safety, and implementation of GDMT among Indian HFrEF patients treated with sacubitril/valsartan therapy.

## Introduction

Heart failure (HF) is a multifaceted clinical syndrome typically resulting in impaired ventricular blood filling or ejection and is estimated to impact about 56.19 million individuals globally [1,2]. It represents a major public health concern due to its high rates of morbidity and mortality [3]. In-hospital mortality rates remain high at 4-6%, while mortality during the three-month vulnerable phase post-discharge is reported at 10-30%. Mortality among chronic HF patients is as high as 50% in five years following diagnosis [4]. The current estimated incidence of HF in India ranges from 0.7 to 1.5 per 1000 persons per year [5]. The age-standardized prevalence rate of HF in India has increased from 390.4 (1990) to 406.2 (2019) per 100,000 population over the last two decades [6]. 

Study rationale

According to data from the Trivandrum Heart Failure Registry, three out of four HF patients present with heart failure with reduced ejection fraction (HFrEF) [7]. Similar findings highlighting the greater prevalence of HFrEF have been reported by some of the Indian HF registries, including the Medanta registry [8], Cardiology Society of India-Kerala Acute Heart Failure Registry (CSI-KHFR) [9], Indian Council of Medical Research National Heart Failure Registry (ICMR-NHFR) [2], and Indian College of Cardiology National Heart Failure Registry (ICC-NHFR) [10].

Over the past three decades, substantial progress has been made in the pharmacological management of HFrEF [11], and guideline-directed medical therapy (GDMT) has emerged as the standard of care for managing HF [12]. Clinical trials have consistently shown the efficacy of GDMT, which includes beta-blockers (BB), renin-angiotensin system antagonists (such as angiotensin-converting enzyme inhibitors (ACEIs), angiotensin receptor blockers (ARBs), and angiotensin receptor-neprilysin inhibitors (ARNIs)), mineralocorticoid receptor antagonists (MRA), and sodium-glucose cotransporter 2 inhibitors (SGLT2i) [13-15]. These therapies have demonstrated improved cardiac function, enhanced quality of life and functional status, and significantly reduced risks of hospitalization and mortality in patients with HFrEF [12]. Thus, the major clinical guidelines for the management of HFrEF strongly recommend the initiation of GDMT [16]. However, according to the Change the Management of Patients with Heart Failure (CHAMP-HF) registry, the implementation of GDMT in routine clinical practice is significantly varied [17]. The Trivandrum Heart Failure Registry data revealed that only 19% of HFrEF patients on admission and 25% of those during discharge were receiving GDMT, raising concerns about adherence to disease-modifying therapy in India compared to prior published cohorts. Data from other Indian HF registries also reflect a similar trend of underutilization of GDMT [2,9,10,18]. Additionally, Indian patients had worse outcomes, with the 90-day mortality rate of 18.1% in the Trivandrum Heart Failure Registry at least twice as high as that of reported mortality rates published elsewhere [7].

Sacubitril-valsartan, the first class of ARNI, has been shown to significantly reduce mortality as well as hospitalizations and rehospitalizations in patients with HFrEF [19]. Although sacubitril-valsartan is recognized as an integral treatment for HF based on clinical trials, there is limited real-world data on its usage and clinical experience, the implementation of GDMT, and the characteristics of HFrEF patients in the Indian context. Additionally, there is limited clarity on the initiation and up-titration regimen of ARNI in routine clinical practice due to concerns about tolerability, particularly the risk of hypotension [20,21].

To address this gap, the Assessing the Dosage Pattern and Demographic Characteristics in Heart Failure Reduced Ejection Fraction Patients Initiated with Angiotensin Receptor-Neprilysin Inhibitor in India (ADD-ARNI) study is a first-of-its-kind prospective, multicenter observational study designed to evaluate the dosage patterns and treatment persistence of ARNI therapy in patients with HFrEF in India.

## Materials and methods

Study design

**Figure 1:**
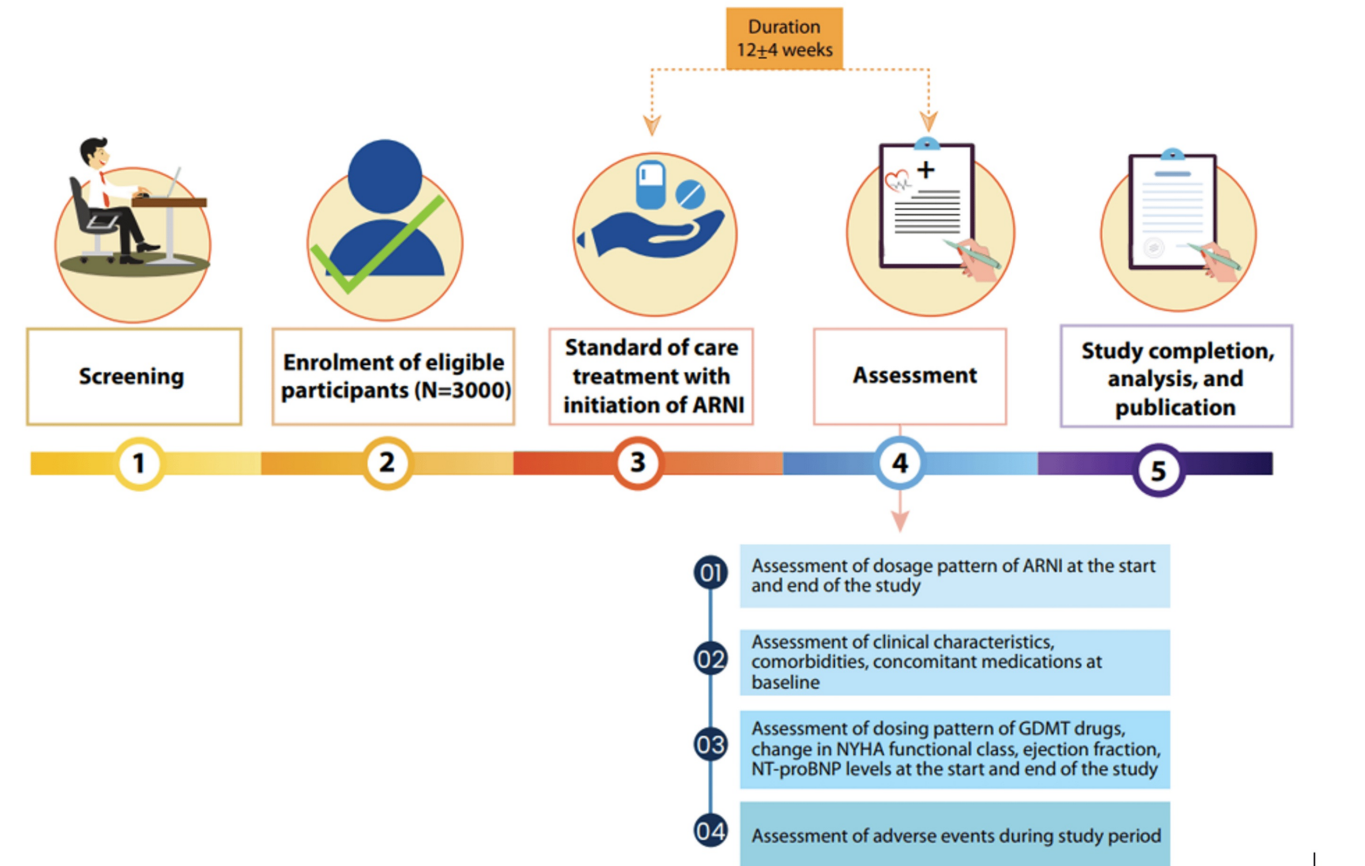
Flowchart of the study ARNI: angiotensin receptor-neprilysin inhibitor; GDMT: guideline-directed medical therapy; NT-proBNP: N-terminal pro-B-type natriuretic peptide; NYHA: New York Heart Association Original figures are created by the authors and are not adapted from any other sources.

The ADD-ARNI study (trial registration number: CTRI/2024/07/071165) is a prospective, multicenter, observational study being conducted at 200 sites across India from September 2024 and is expected to be completed by June 2025. The primary objective of this study is to assess the dosage pattern and treatment persistence of ARNI therapy in patients with HFrEF in India. The secondary objectives are to describe the medication dosage pattern and the implementation of GDMT in patients with HFrEF initiated on ARNI therapy and to evaluate its safety and effectiveness. This study was approved by the Suraksha Institutional Ethics Committee (approval number: N/A) and is being conducted in accordance with the principles of the Declaration of Helsinki, Indian Council of Medical Research (ICMR), International Conference on Harmonization (ICH), Good Clinical Practice (GCP) guidelines, and applicable legislations. The overall study design of the ADD-ARNI study is depicted in Figure 1.

Study population

The study population will consist of symptomatic chronic HFrEF patients as per the European Society of Cardiology (ESC) 2016 criteria [22] and who are eligible for the initiation of sacubitril/valsartan tablets as per the judgement of the treating physician. Patients who are recently diagnosed with HFrEF or chronic HFrEF patients with left ventricular ejection fraction (LVEF) ≤40% and documented for not more than three months, who are stable, and who have not been hospitalized for the past one month prior to enrolment in the study shall be included. Written informed consent shall be obtained from all participants prior to their inclusion in the study. Patients who were deemed ineligible included pregnant and lactating women, patients with acute decompensated HF, patients with hyperkalemia (serum potassium level ≥5.30 mmol/L) within the past two weeks, and patients participating in any other clinical study. Patients should meet all the inclusion criteria and none of the exclusion criteria prior to being enrolled in the study.

Study treatment, duration, and endpoints

Study participants who meet all the inclusion criteria and are deemed eligible for the initiation of ARNI therapy as per the clinical judgement of the treating physician will be initiated on ARNI therapy as per routine clinical practice. This is an observational study that will be conducted over a period of 12 weeks±4 weeks from the date of enrolment of the patient.

The primary endpoint of the study is the dosage pattern of ARNI at the start and end of the study. The secondary endpoints include the HFrEF medication dosing pattern at the start and end of the study with regard to the doses of BB, MRA, SGLT2i, and diuretics, the implementation of GDMT at the end of the study, the assessment of the effectiveness of ARNI therapy based on changes in New York Heart Association (NYHA) functional class and N-terminal pro-B-type natriuretic peptide (NT-proBNP) levels from baseline to follow-up, and the incidence of adverse events, either spontaneously reported by the patients or noticed by the clinician during the study.

Data collection

All data required to fulfill the primary and secondary objectives of the study will be captured in an electronic data capture platform. The data related to demographic and clinical characteristics of patients will be collected at baseline. Additionally, the data points with respect to clinical parameters such as vitals, estimated glomerular filtration rate (eGFR), serum creatinine, serum potassium, and NT-proBNP will be recorded at baseline. Medication-related data, including type of medications, dosage, and titration, will be recorded at baseline and at the end of 12±4 weeks. Any incidences of adverse events will be documented during the study period.

Safety reporting

All adverse events will be captured starting from the date of enrolment until the end of the study, regardless of whether or not they are related to the study intervention. Adverse events, not limited to symptomatic hypotension (systolic blood pressure (SBP) ≤100 mmHg with symptoms like dizziness, blurry vision, fainting, fatigue, and unsteadiness), asymptomatic hypotension (SBP ≤90 mmHg with no symptoms), hyperkalemia (serum potassium levels >5.5 mmol/L), impaired renal function (decline in the eGFR of ≥50%), and persistent cough, shall be recorded during follow-up visits along with any additional adverse events [14]. It is the responsibility of the investigator to capture and report the adverse events (serious and non-serious).

Sample size calculation

The list of potential demographic and clinical characteristics, along with the distributions of patients with HFrEF, was extracted from Philip et al. [23] and Pokharel et al. [24]. Expecting a similar distribution of these characteristics in the current study, the minimum required sample sizes were calculated for 5%, 3%, and 2% precision with a 95% confidence interval (CI). In this study, the target patient population is 3,000, and accounting for an estimated ~15% attrition rate, the sample size is set at 3,600.

The following formulas were used for sample size calculation: for prevalence, $n=\frac{\left(1.96\right)^{2}p\left(1-p\right)}{d^{2}}$, while for mean, $n=\frac{\left(1.96xSD\right)^{2}}{d^{2}}$. Here, p means proportion or prevalence; SD, standard deviation; and d, precision. For proportion, d is absolute, but for mean, it is relative to the mean.

Statistical consideration

All statistical tests will be performed using appropriate software, with a two-tailed overall significance level of 0.05. Categorical data (counts and percentages) will be compared between baseline and 12 weeks using the chi-squared test (or any other test deemed appropriate), while numerical data will be compared between the two groups using an unpaired t-test (or any other test deemed appropriate).

## Results

The expected results of the study will include the dosage pattern of ARNI therapy at both the start and end of the study, the dosing patterns of other HFrEF medications, the implementation of GDMT, and the effectiveness of ARNI therapy as assessed by changes in NYHA functional class and NT-proBNP levels from baseline to follow-up. Additionally, the incidence of adverse events will be recorded.

## Discussion

The primary objective of HF treatment is to effectively prevent complications while ensuring prolonged survival, free from morbidity [11]. ADD-ARNI is the first-of-its-kind prospective, multicenter observational study designed to assess the dosage patterns and treatment persistence of ARNI therapy in patients with HFrEF in India. This study also seeks to analyze the implementation of GDMT, the effectiveness of ARNI therapy in terms of changes in NYHA functional class and NT-proBNP levels, functional improvements, and adverse events. These findings are expected to provide insights into the real-world use of ARNI and its impact on HF management in the Indian population.

Data from Indian HF registries suggest a relatively higher prevalence of HFrEF (Table 1) [2,8-10,18].

**Table 1:** Trends in Indian HF-EF distribution N=total number of patients for each registry CSI-Kerala: Cardiology Society of India-Kerala Acute Heart Failure Registry; ICC-NHFR: Indian College of Cardiology National Heart Failure Registry; ICMR-NHFR: Indian Council of Medical Research National Heart Failure Registry; THFR: Trivandrum Heart Failure Registry; HFrEF: heart failure with reduced ejection fraction; LVEF: left ventricular ejection fraction; HFpEF: heart failure with preserved ejection fraction; HFmEF: heart failure with mildly reduced ejection fraction; HFrEF: heart failure with reduced ejection fraction The data for HFpEF and HFmEF from the Medanta and ICC-NHFR registries have been presented in a combined format, as reported in the original sources.

Registry	HFrEF	HFpEF	HFmEF
THFR (n=1205) [18]	62%	20%	18%
CSI-Kerala (n=7507) [9]	65.7%	14.9%	17.6%
Medanta (n=5590) [8]	59.1% (LVEF ≤30%)	41.9% (LVEF ≥30%)	
ICC-NHFR (n=5269) [10]	68.29% (LVEF <40%)	31.7 1 (LVEF >40%)	
ICMR-NHFR (n=10851) [2]	65.2%	12.6%	22%

**Figure 2:**
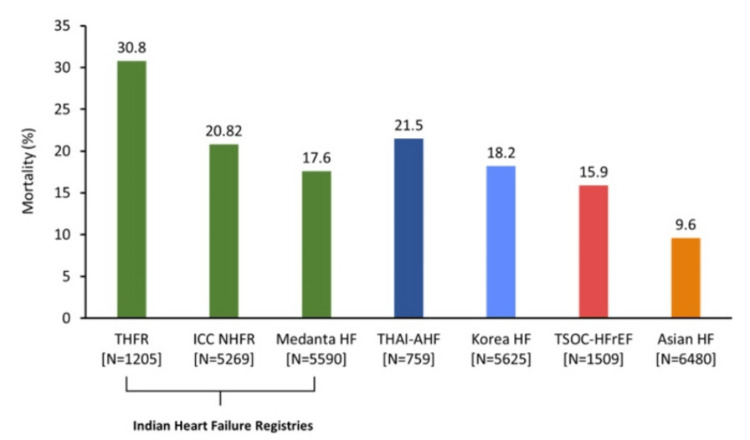
One-year mortality rates in Asia N=total number of patients for each registry Asian HF: Asian heart failure [28]; ICC-NHFR: Indian College of Cardiology National Heart Failure Registry [10]; Korea HF: Korea heart failure [29]; Medanta HF: Medanta heart failure [8]; THAI-AHF: Thailand-acute heart failure [26]; THFR: Trivandrum Heart Failure Registry [18]; TSOC-HFrEF: Taiwan Society of Cardiology-heart failure with reduced ejection fraction [27] Original figures are created by the authors and are not adapted from any other sources.

HFrEF poses a significant public health challenge due to its associated high morbidity and mortality rates [25]. In India, the one-year mortality rate is relatively higher at 31% compared to other Asian countries (Figure 2) [8,10,18,26-29].

For patients with HFrEF, adhering to guideline-recommended therapies and ensuring up-titration to evidence-based doses is a proven strategy to deliver optimal care. This approach plays a critical role in reducing the risk of recurrent HF hospitalizations and cardiovascular mortality [16,30]. The present study will assess persistence with ARNI therapy over a 12-week period, explore the titration to target doses, and identify barriers to achieving these targets.

Evidence from previous studies, such as the CHAMP-HF Registry, evaluated the effectiveness of medical therapy dosing for HF in outpatients with chronic HFrEF. The study found that compared to target dosing, lower dosages and non-use of ACEI/ARB/ARNI and beta-blocker therapies were independently associated with higher mortality rates. Additionally, lower doses of ACEIs/ARBs/ARNIs were associated with an increased risk of HF hospitalizations and a higher five-year mortality [17].

**Figure 3:**
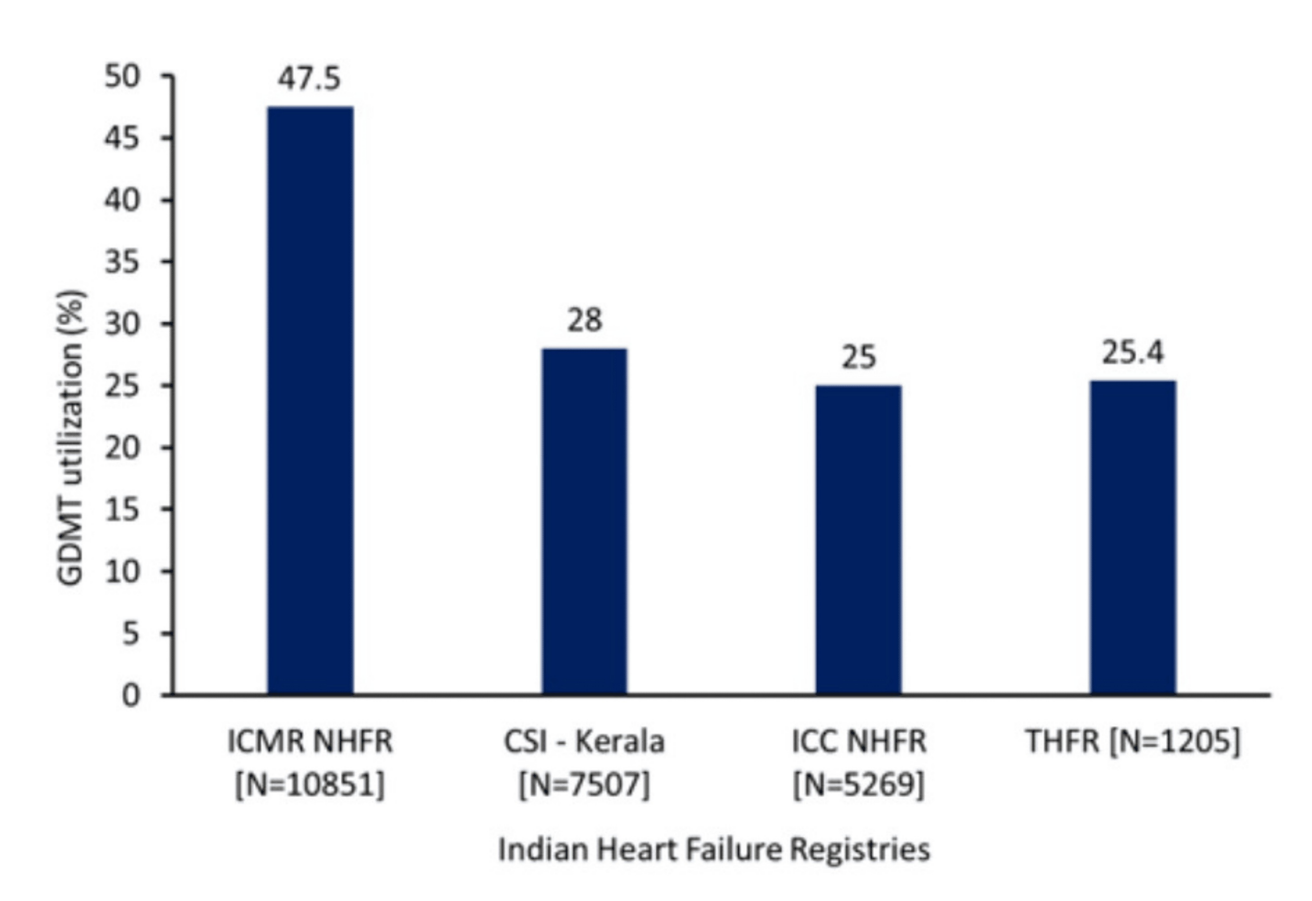
Implementation of GDMT in India N=total number of patients for each registry CSI-Kerala: Cardiology Society of India-Kerala Acute Heart Failure Registry [9]; GDMT: guideline-directed medical therapy; ICC-NHFR: Indian College of Cardiology National Heart Failure Registry [10]; ICMR-NHFR: Indian Council of Medical Research National Heart Failure Registry [2]; THFR: Trivandrum Heart Failure Registry [18] Original figures are created by the authors and are not adapted from any other sources.

The secondary objective of the present study will be to evaluate the implementation of GDMT at the end of the study, expressed as the proportion of patients on two, three, or four GDMT drugs. Previous studies, including the ICMR-NHFR study, reported that nearly one in two eligible patients with HF received GDMT. The importance of GDMT lies in its association with improved survival rates for patients with HFrEF and heart failure with mildly reduced ejection fraction (HFmrEF). In contrast, those with HFrEF and HFmrEF who did not receive GDMT experienced higher mortality rates, underlining the critical role of GDMT in improving outcomes. The overall 90-day mortality rate for these patients was reported as 14.2% [2]. Furthermore, the Trivandrum Heart Failure Registry has highlighted significant gaps in the implementation of GDMT, despite its proven association with reduced mortality and improved survival. In the Trivandrum Heart Failure Registry, for instance, only one in six patients received guideline-based in-hospital treatment, which was associated with improved survival rates and lower mortality [7]. The present study aims to explore whether similar trends exist in its cohort, contributing further insights into the gaps in GDMT adherence. Additionally, data from the CSI-KHFR provide further evidence of suboptimal GDMT implementation, as only one in four patients received guideline-based treatment (Figure 3).

The CSI-KHFR also reported an in-hospital mortality rate of 7% and a 90-day mortality rate of 11.6% [9]. These findings highlight the critical need for improving GDMT adherence in the management of HF in India.

In the present study, the effectiveness of ARNI therapy will be evaluated based on changes in NYHA functional class and NT-proBNP levels from baseline to follow-up. Previous findings suggest that improvement in the NYHA class is associated with a lower risk of all-cause and cardiovascular mortality, as well as a reduced likelihood of first HF hospitalization [31]. Furthermore, another study reported that lower NT-proBNP levels are associated with a reduced six-month all-cause mortality rate [32].

Previous studies have demonstrated the efficacy of ARNI in improving both NYHA class and NT-proBNP levels [33-35]. The PARADIGM-HF India sub-study specifically highlighted the effectiveness of ARNI in improving NYHA class compared to enalapril [33]. Similarly, a single-center, open-label observational registry of consecutive HF patients initiated on ARNI at the National Heart Centre Singapore found that ARNI resulted in improvements in both NYHA class and NT-proBNP levels at three months compared to baseline and was generally well-tolerated [34]. The PROVE-HF trial, a prospective study of biomarkers, symptom improvement, and ventricular remodeling during sacubitril-valsartan therapy for HF, found that patients with HFrEF treated with sacubitril-valsartan experienced a rapid reduction in NT-proBNP, a well-established biomarker for HF severity and prognosis. This reduction was weakly but significantly correlated with improvements in markers of cardiac volume and function at 12 months, suggesting reverse cardiac remodeling after one year [35].

Despite the growing body of evidence supporting the benefits of sacubitril-valsartan therapy in clinical trials, there remains a paucity of real-world data regarding its use, the utilization of GDMT, and insights from clinical experience in India, particularly concerning the characteristics of HFrEF patients treated in routine clinical practice. This lack of data is especially concerning given the unique demographic and clinical characteristics of the Indian population, which could influence treatment outcomes. Therefore, a real-world study is essential to evaluate the use of sacubitril-valsartan therapy and the optimal implementation of GDMT in India. The ADD-ARNI study seeks to bridge this gap by providing evidence-based insights into the effectiveness of ARNI therapy in an Indian cohort. The findings of this study could significantly impact clinical decision-making, ultimately helping to optimize treatment strategies and improve patient outcomes in India.

The present study will also monitor adverse events associated with ARNI therapy, either spontaneously reported by patients or identified by clinicians. The PARADIGM-HF India sub-study reported that the most common adverse events (>10%) were cough, hyperkalemia, hypotension, and dyspnea [33]. Likewise, a retrospective cohort study conducted by Koçak et al. reported that hypotension was the most frequently observed adverse event, affecting 16% of patients with HF initiated on sacubitril-valsartan; among these, 4% were symptomatic [36]. A previous real-world study in India included patients aged ≥18 years with HFrEF (LVEF <40%) who were prescribed sacubitril-valsartan, assessing its dose titration over six months [37]. In contrast, the present study will evaluate the overall dosage patterns of ARNI therapy and the implementation of GDMT for HF, aiming to provide a broader perspective on real-world practice in India.

Strengths and limitations

This study has several notable strengths. ADD-ARNI is the first-of-its-kind prospective, multicenter observational study designed to assess the dosage patterns and treatment persistence of ARNI therapy specifically in patients with HFrEF in India. By including a substantial HFrEF population, the study ensures a more comprehensive representation of patients across diverse real-world settings. Furthermore, it addresses the existing gap in literature by providing evidence on the effectiveness of ARNI therapy in an Indian cohort, a population in which limited real-world studies have been conducted. Additionally, this study will provide valuable insights into dosage patterns, treatment persistence, and the implementation of GDMT in patients with HFrEF initiated on ARNI therapy, providing practical data to inform clinical decision-making.

Despite its strengths, the study has some limitations. The shorter duration of 12 weeks may not be sufficient to capture long-term treatment persistence, adherence, and outcomes associated with ARNI therapy. While a longer duration could have provided more robust data on the sustained effectiveness and safety of ARNI therapy, it also comes with certain constraints, such as increased resource requirements and logistical challenges. Therefore, the present study opted for a shorter duration to ensure feasibility and timely completion. Additionally, as a non-randomized, real-world study, it is inherently prone to bias, which could influence the results. Factors such as variations in clinician practices, patient characteristics, and adherence to therapy may contribute to this bias. By acknowledging these limitations, the study highlights areas for future research to build upon its findings.

## Conclusions

The ADD-ARNI study is designed to evaluate dosage patterns, treatment persistence, and the implementation of GDMT in patients with HFrEF initiated on ARNI therapy in India. This real-world study will provide critical insights into the use of ARNI and associated outcomes in Indian patients with HFrEF. Given the limited real-world data on the use of ARNI, the implementation of GDMT, and clinical evidence for sacubitril-valsartan therapy in the Indian population, this study seeks to bridge the gap by providing evidence-based insights into the effectiveness of sacubitril-valsartan therapy in an Indian cohort and improve the adoption of evidence-based pharmacotherapy in India.
